# Time-Resolved Metabolomics Reveals Mitochondrial Protection in Septic Liver Injury

**DOI:** 10.3390/metabo15090600

**Published:** 2025-09-09

**Authors:** Naoki Suzuki, Shoichiro Shibata, Masahiro Sugimoto, Eskil Elmer, Hiroyuki Uchino

**Affiliations:** 1 Department of Anesthesiology, Tokyo Medical University, Tokyo 1600023, Japan; suzuki-n@tokyo-med.ac.jp (N.S.);; 2 Institute for Advanced Biosciences, Keio University, Tsuruoka 9970052, Japan; msugi@sfc.keio.ac.jp; 3 Institute of Medical Sciences, Tokyo Medical University, Tokyo 1600023, Japan; 4 Department of Clinical Science, Lund University, 22184 Lund, Sweden

**Keywords:** metabolomics, sepsis, liver injury, mitochondrial dysfunction, cyclophilin D, mitochondrial permeability transition, mouse

## Abstract

**Background/Objectives**: Sepsis is a life-threatening condition characterized by organ dysfunction due to a dysregulated host response to infection. Mitochondrial dysfunction is considered a key contributor to the pathogenesis of sepsis, but its molecular mechanisms remain unclear. **Methods**: In this study, we used a cecal ligation and puncture (CLP) model to induce sepsis in wild-type (WT) and cyclophilin D knockout (CypD KO) mice. Liver tissues were collected at 0, 6, and 18 h post-CLP and analyzed using liquid chromatography–tandem mass spectrometry (LC-MS/MS). **Results**: Metabolomic profiling revealed that lactate levels significantly increased in the WT mice but remained stable in the KO mice. While AMP levels were preserved in the KO mice, these mice had significantly higher glutathione disulfide (GSSG) and spermidine concentrations than the WT mice at 18 h (*p* < 0.05). The levels of malondialdehyde (MDA), a marker of oxidative stress, were also significantly lower in the KO mice at 18 h (*p* < 0.05). These findings suggest that CypD deficiency preserves mitochondrial function, enhances resistance to oxidative stress, and mitigates septic liver injury. **Conclusions:** Our results highlight the potential of targeting mitochondrial permeability transition as a therapeutic strategy for sepsis.

## 1. Introduction

Sepsis is a life-threatening condition characterized by organ dysfunction resulting from a dysregulated host response to infection [1,2]. Despite advances in intensive care, sepsis remains a major cause of mortality worldwide, with liver dysfunction being a critical determinant of poor prognosis [2]. The liver plays a central role in immune regulation, detoxification, and metabolic homeostasis during systemic inflammation. However, excessive inflammatory responses and microcirculatory disturbances can lead to hepatocellular injury, mitochondrial dysfunction, and ultimately liver failure. These observations highlight the importance of elucidating the molecular mechanisms underlying septic liver injury, thereby enhancing therapeutic strategies.

Mitochondrial dysfunction has emerged as a key contributor to multiple organ failure in sepsis [3,4,5]. Specifically, the mitochondrial permeability transition (MPT) plays a crucial role in mitochondrial dysfunction. The MPT is a Ca^2+^-dependent and cyclophilin D (CypD)-facilitated increase in the degree of openness of the inner mitochondrial permeability transition pore (MPTP), allowing for the diffusion of molecules up to 1.5 kDa in size [6]. Studies have shown that the opening of the MPTP leads to a loss of membrane potential, ATP depletion, and cell death [7]. Cyclophilin D (CypD), a peptidyl-prolyl isomerase located in the mitochondrial matrix, is a critical regulator of MPTP opening [8]. Previous work performed by our group demonstrated that CypD knockout (CypD KO) mice and those treat with cyclosporin A (CsA) exhibit improved thermoregulation, reduced septic encephalopathy, ameliorated ischemic brain damage and traumatic brain injury [9,10,11,12], and enhanced survival following sepsis induction [4]. However, the precise metabolic consequences of CypD deletion in peripheral organs such as the liver remain poorly understood. Thus, a deeper understanding of how CypD modulates hepatic metabolism, the production of oxidative stress, and cell survival under septic conditions is required.

Recent advances in metabolomics have enabled comprehensive profiling of small-molecule metabolites, offering a powerful approach to investigating mitochondrial function and redox balance in disease states [13]. In particular, mass-spectrometry-based metabolomics allows for high-resolution analysis of polar metabolites involved in energy metabolism, oxidative stress, and amino acid turnover. Several studies have applied metabolomics to sepsis models, revealing alterations in glycolysis, the TCA cycle, and glutathione metabolism. For example, Liang et al. used lipidomics to identify key lipid mediators in septic liver injury [14], while Huang et al. combined transcriptomics and metabolomics to elucidate the protective effects of agmatine in septic rats [15]. These findings highlight the utility of metabolomics in uncovering metabolic signatures and potential therapeutic targets in regard to sepsis.

Despite these advances, few studies have directly examined the metabolic impact of MPTP inhibition or CypD deletion in the context of septic liver injury [2]. Moreover, the temporal dynamics of metabolite changes during sepsis progression remain largely unexplored. In this study, we employed LC-TOFMS-based metabolomics to analyze liver tissues from wild-type and CypD KO mice subjected to cecal ligation and puncture (CLP), a clinically relevant model of polymicrobial sepsis. By integrating metabolic profiling with oxidative stress markers and survival data, we aimed to clarify the role of CypD in hepatic mitochondrial resilience and identify key metabolic pathways associated with sepsis resistance.

## 2. Materials and Methods

### 2.1. Sepsis Model

The design of this study was approved by the Tokyo Medical University Animal Experiment Committee (Approval No: R5-136, approved on 8 November 2023), and all procedures were conducted in accordance with institutional and national guidelines for animal experimentation. The sepsis model mice were established according to the method previously reported by Kobayashi et al. (2022) [4]. Male C57BL/6 wild-type (WT) mice (10–16 weeks old, purchased from CLEA Tokyo Japan, Inc.) and male CypD knockout (Ppif^−/−^) mice were used. CypD knockout (Ppif^−/−^) mice were generated by breeding and genotyping of CypD heterozygous mice (The Jackson Laboratory, Bar Harbor, ME, USA, #022308 B6;129-Ppif<tm1Maf>/J). A total of 27 WT mice and 27 CypD KO mice (9 mice for metabolomics, 18 mice for thiobarbituric acid reactive substances (TBARS) assay and Western blot analysis, respectively) were included in the study. All animals were housed in temperature-controlled rooms (24–28 °C) under a 12-h light–dark cycle (lights on at 7:00 a.m., lights off at 7:00 p.m.) and had free access to food and water. Five mice were housed per cage. Environmental enrichment was provided, and the sample size was determined based on previous studies using similar animal models. To induce sepsis, mice were anesthetized with 4–5% sevoflurane, and a midline abdominal incision was made to expose the cecum. Subsequently, the cecal ligation and perforation (CLP) procedure was performed. After surgery, the animals were kept under minimal heating, with free access to food and water, and were monitored according to the following protocol. The well-being assessment was conducted over an 18-h period by observing the animals at regular intervals via a remote camera. The endpoints were defined as when the body temperature fell below 26 degrees or 18 h after surgery. Animals that reached the endpoint were euthanized by decapitation after being anesthetized with 8% sevoflurane. Experimental animals were excluded from the analysis only if they died during the observation period, and all surviving animals were included in the analysis. The WT mice group and the CypD KO mice group were randomly assigned to three time points (0 h, 6 h, and 18 h after CLP as in Kobayashi et al. [4]). At the assigned time points, mice were anesthetized with 4–5% sevoflurane, and a midline abdominal incision was made to collect the liver tissue. After harvesting, the liver tissue was immediately frozen in liquid nitrogen, and the mice were sacrificed. Group assignment, experimental procedures, evaluation of results, and data analysis were conducted with Dr. Suzuki aware of the group assignments.

### 2.2. Metabolomic Analyses

#### 2.2.1. Chemicals

Liquid chromatography–tandem mass spectrometry (LC-MS)-grade methanol and formic acid (FA) were obtained from FUJIFILM Wako (Osaka, Japan), along with a 28% (*w*/*w*) ammonia solution. Heptafluorobutyric acid (approximately 0.5 mol/L in water) was purchased from TCI (Tokyo, Japan). Eleven reagents were used as internal standards (ISs). Spermidine-d8, spermine-d8, and methionine sulfone were obtained from Sigma Aldrich (St. Louis, MO, USA). N1-Acetylspermidine-d6, N1, N12-diacetylspermine-d6, N1, N8-diacetylspermidine-d6, N1-Acetylspermine-d3, and hypoxanthine-13C2,15N were obtained from Santa Cruz (Dallas, TX, USA). Camphor-10-sulfonic acid and Sulfanilic acid were acquired from FUJIFILM Wako. 1,6-Diaminohexane was obtained from Tokyo Chemical Industry Co., Ltd. (Tokyo, Japan). Water was purified using a Milli-Q system (Merck Millipore, Bedford, MA, USA).

#### 2.2.2. Pretreatment of Tissue Samples

For metabolite extraction, frozen tissue samples (approximately 50 mg) were plunged into a Precellys CKmix lysing tube (Bertin Technologies, Montigny-le-Bretonneux, France) containing 0.5 mL of methanol. The samples were homogenized by vibrating them 3 times (6500 rpm for 20 s, followed by a 50 s pause) at 4 °C. The homogenate (40 mL) was mixed with methanol (80 mL) containing 1.5 mM internal standards (camphor-10-sulfonic acid) and 0.15 mM internal standards (d8-spermine, d8-spermidine, d6-N1-acetylspermidine, d3-N1-Acetylspermine, d6-N1, N8-diacetylspermidine, d6-N1, N12-diacetylspermine, hypoxanthine-13C,15N, and 1,6-diaminohexane). After centrifugation at 20,380× *g* for 10 min at 4 °C, the entire supernatant was transferred to another tube and vacuum-dried. The sample was reconstituted with 90% methanol (*v*/*v*, 16 mL) and water (48 mL) and then vortexed and centrifuged at 20,380× *g* for 10 min at 4 °C. The supernatants were injected into an LC-MS/MS system.

#### 2.2.3. LC Condition

The HPLC system used was an Agilent 1290 Infinity LC system (Agilent Technologies, Santa Clara, CA, USA). Analytic separation of cationic metabolites was achieved using a Waters Acquity BEH C18 column (2.1 i.d. × 50 mm, 1.7 mm; Waters, Milford, MA, USA) at 40 °C. The mobile phase, consisting of solvent A (0.1% Formic acid and 1.5 mM HFBA in water) and solvent B (1.5 mM HFBA in methanol), was delivered at a flow rate of 0.3 mL/min. The gradient elution conditions of pump A were 99% at 0.00 min, 99% at 0.60 min, 58% at 0.80 min, 58% at 1.80 min, 50% at 2.30 min, 50% at 3.00 min, 5% at 4.00 min, and 5% at 6.00 min. The run time was 11 min per sample. For the anionic metabolites, chromatographic separation was performed using an ACQUITY HSS T3 column (2.1 i.d. × 50 mm, 1.8 mm; Waters, Milford, MA, USA) at 30 °C. The mobile phase, consisting of solvent A (0.1% Formic acid in water) and solvent B (Acetonitrile), was delivered at a flow rate of 0.3 mL/min. The gradient elution conditions of pump A were 100% at 0.00 min, 100% at 1.00 min, 95% at 1.01 min, 95% at 1.50 min, 70% at 1.51 min, 70% at 2.50 min, 40% at 2.51 min, 40% at 3.50 min, 15% at 3.51 min, 15% at 4.00 min, 5% at 4.01 min, and 5% at 6.00 min. The run time was 11 min per sample. 

#### 2.2.4. MS Conditions

Mass spectrometry was performed in positive ionization mode for cationic metabolites and negative ionization mode for anionic metabolites using an Agilent Technologies G6230B time-of-flight MS (TOF-MS). Instrument parameters were set as follows: drying gas temperature, 350 °C; drying gas flow, 13 L/min; nebulizer, 55 psig; Vcap, 3500 V; fragmentor, 125 V; Skimmer1, 90 V; octopoleRFPeak, 200 V, mass range, 50–1000 *m*/*z* and 50–1200 *m*/*z* for positive and negative mode, respectively; and scan rate, 1.00 spectra/s. Agilent Mass Hunter Qualitative Analysis and Quantitative Analysis software were used for data processing (version B.08.00, Agilent Technologies).

#### 2.2.5. Data Processing

Raw data were analyzed using MassHunter Workstation Software Quantitative Analysis (ver. B.08.00; Agilent Technologies). Metabolome analysis was conducted on the processed data. To eliminate unexpected bias, the sequence of samples was randomized, and the standard mixture and quality control (QC) samples were also measured. The QC samples among the nine samples were measured. The total ion chromatography results for each sample were compared to those for the QC samples: we observed that the similarity of the background was <10%. The QC samples were mixed with methanol extracts of mouse cerebral cortex, liver, and kidney tissue and then pretreated as described for the tissue samples. We confirmed the inclusion of the internal standard and acceptable errors in the intensity of the internal standard, as specified.

Additionally, we verified that the errors in the intensity and *m*/*z* (<10 ppm) and retention time (<0.1 min) of the internal standard were acceptable. We confirmed the relative area (i.e., the peak area of each metabolite divided by the area of the internal standard) and noted the small relative deviation of fluctuation (<20%) of the detected peaks in the QC samples. We prepared a standard mixture containing 104 and 45 metabolites for the positive and negative modes, respectively, and established the linearity range of each metabolite before analyzing the samples.

The peaks on the extracted chromatograms of the standard mixture were analyzed first, followed by the peaks corresponding to the standard mixture in the samples. The absolute concentrations of tissue metabolites were calculated based on the ratio of the relative peak area in each sample to the standard mixture. Subsequently, the concentration was divided by the weight of the sample (in millimoles per gram of wet weight). The peaks whose peak size was less than the lower limit of linearity were considered non-detected. The peaks with insufficient separation were treated as single peaks. The frequently detected peaks (present in 50% of the samples) were used for statistical analyses.

### 2.3. Measurement of MDA via TBARS Assay

Malondialdehyde (MDA), a marker of oxidative stress, was quantified using the thiobarbituric acid reactive substances (TBARS) assay. Measurements were performed according to the manufacturer’s instructions for the TBARS assay kit (Cayman Chemical, Ann Arbor, MI, USA, #10009055). Briefly, 25 μg of mouse liver tissue was homogenized in 250 μL of RIPA buffer (Santa Cruz Biotechnology, Santa Cruz, CA, USA) containing 2 mM phenylmethylsulfonyl fluoride (PMSF), 1 mM sodium orthovanadate, and protease inhibitor cocktail and centrifuged at 1600× *g* for 10 min. Then, the supernatant was mixed with the color reagent. The mixture was heated at 95 °C for 60 min, cooled, and centrifuged. The absorbance of the supernatant was measured at 532 nm using a spectrophotometer. The MDA concentration was calculated using a colorimetric standard curve prepared using MDA.

### 2.4. Western Blot Analysis

Liver tissue samples were collected at 0 h, 6 h, and 18 h after the CLP procedure (*n* = 6 per group). Samples were extracted on ice in RIPA buffer (Sant Cruz Biotechnology) containing 2 mM PMSF, 1 mM sodium orthovanadate, and a protease inhibitor cocktail and then centrifuged (10,000× *g*) for 15 min at 4 °C. The supernatant was diluted in Laemmli sample buffer containing 2-mercaptoethanol (Bio-Rad Laboratories, Hercules, CA, USA, #161-0737) and heated at 100 °C for 10 min. Samples containing 30 μg of protein per lane each were loaded onto a 12% precast gel (Bio-Rad Laboratories, #4561046) along with molecular-weight standards and electrophoresed for 60 min at 100 V. Proteins separated via 12% SDS-PAGE were transferred to a PVDF membrane via electroblotting at 15 V for 3 min using the Trans-Blot Turbo Transfer System (Bio-Rad Laboratories, Hercules, CA, USA), filter paper, and PVDF membranes, along with the Trans-Blot Turbo Transfer Pack. The proteins that had been separated on 12% SDS–PAGE were transferred to PVDF membranes via electroblotting at 15 V for 3 min. After blocking, the membranes were incubated with anti-phospho-Akt (Ser473) (D9E) rabbit monoclonal antibody (Cell Signaling Technology, Danvers, MA, USA, #4060S), anti-Akt (pan) (11E7) rabbit monoclonal antibody (Cell Signaling Technology, Danvers, MA, USA, #4685S), or anti-beta-Actin mouse monoclonal antibody (Abcam, Cambridge, Cambridgeshire, UK, ab8226) in Block Solution overnight at 4 °C. The membranes were then washed three times in Tris-buffered saline with 0.05% Tween 20 (TBS-T) and incubated with peroxidase-labeled anti-rabbit IgG(H + L) antibody (SeraCare Life Sciences, Milford, MA, USA, #5450-0010) or peroxidase-labeled anti-mouse IgG(H + L) antibody (SeraCare Life Sciences, #5450-0011) for 1 h at room temperature. Subsequently, the membranes were washed with TBS-T, and protein bands were detected with ECL Select Western Blotting Detection Reagent (GE Healthcare Life Sciences, Chicago, IL, USA, #RPN2235). Molecular-weight standards were used to determine the molecular weights of immunoreactive species. Densitometry was performed using a Molecular Imager ChemiDoc XRS Plus System (Bio-Rad Laboratories). P-Akt is a marker of cell survival signals. The resulting signals were quantified, and the phospho-Akt (P-Akt)/Akt (pan) ratio was calculated and evaluated.

### 2.5. Statistical Analysis

We created a heatmap and conducted principal component analysis using MetaboAnalyst (ver. 6, https://www.metaboanalyst.ca/). The data are expressed as means ± SD. Statistical analysis was performed using Excel andMetaboAnalyst. A comparison of three or more datasets was performed using a one-way analysis of variance (one-way ANOVA). The Student’s *t*-test was used to compare the two groups. Differences were considered statistically significant when the *p*-value was less than 0.05.

## 3. Results

### 3.1. Overview of the Metabolomic Profile

**Figure 1:**
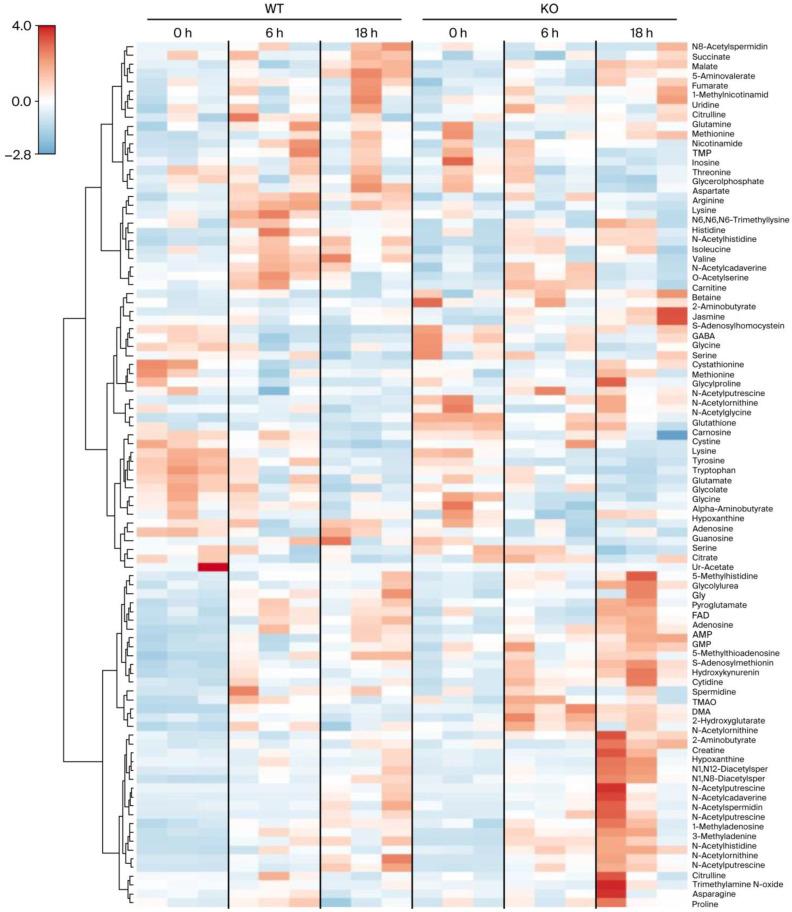
A heatmap of the metabolite concentration profiles in the liver tissue. Each row represents a metabolite, and each column corresponds to a tissue sample. Colors indicate Z-score-normalized values for each metabolite, using a red–white–blue color scale: red denotes relatively high concentrations, while blue indicates relatively low concentrations. Metabolites are clustered based on elucidation distance, so those with similar concentration patterns are positioned adjacent to each other. The data comprise six experimental groups: the wild-type (WT) and knockout (KO) mice 0, 6, and 18 h after CLP. Each group includes triplicate samples. The raw data are provided in Supplementary Materials, Table S1.

A heatmap of liver metabolite concentrations was generated using LC-TOFMS data obtained from the WT and CypD KO mice 0, 6, and 18 h after CLP treatment (Figure 1). Each row represents a metabolite, and each column corresponds to an individual sample. The three adjacent columns, separated by black vertical lines, represent biological triplicates under the same conditions. The consistent color patterns within each triplicate group indicate the high reproducibility of the metabolomic measurements.

In the WT mice, the metabolic profile at 0 h differs noticeably from that at 6 and 18 h. For example, at 0 h, many metabolites with high relative concentrations (red) are located in the central region of the heatmap, whereas at 6 and 18 h, the high-concentration metabolites shift toward the upper region. These data suggest a time-dependent reorganization of metabolic activity following the induction of sepsis.

In contrast, the KO mice showed a progressive shift in metabolic profiles over time. From 0 to 6 h, moderate changes were observed, but by 18 h, a more pronounced alteration emerged. Notably, at 18 h, many metabolites with elevated concentrations are located in the lower portion of the heat map, indicating a distinct metabolic response compared to that of the WT mice. These findings suggest that CypD deficiency alters the temporal dynamics of hepatic metabolism during the progression of sepsis.

### 3.2. Differences in Metabolomic Profiles Between WT and KO

**Figure 2:**
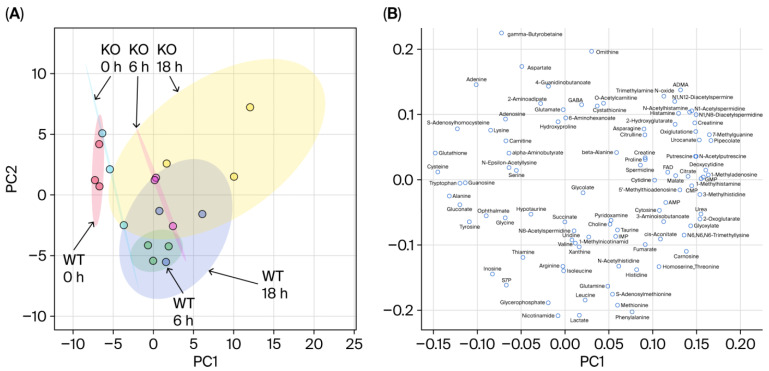
Results of the principal component analysis (PCA). (**A**) Score plots: each point represents one sample. The distance between points reflects the similarity of metabolite concentration patterns across samples; closer points indicate more similar profiles. Ellipses represent the 95% confidence intervals for each group. (**B**) Loading plots: each point represents a metabolite. For example, ornithine appears in the upper central region, indicating that samples located in the upper region of the score plot—i.e., those with higher PC2 values—tend to have relatively higher concentrations of ornithine. PCA was performed using Z-score-normalized metabolite values. The contribution rates of principal component 1 (PC1) and principal component 2 (PC2) are 31.6% and 13.6%, respectively.

Figure 2 presents the results of a principal component analysis (PCA) conducted to visualize the metabolic differences between the WT and KO mice over time. The left panel shows the score plots (Figure 2A). Both the WT and KO groups exhibit a general shift from left to right along the principal component axis as time progresses from 0 h to 6 h and 18 h, indicating time-dependent metabolic changes. At 0 h, the WT and KO samples largely overlap, suggesting that their metabolic profiles were initially similar. The 0 h samples are tightly clustered, reflecting low metabolic diversity at baseline. The WT samples at 6 h and 18 h remain relatively compact, indicating less variability within the group. In contrast, the KO samples at 18 h are widely dispersed, suggesting increased metabolic diversity and a more heterogeneous response.

The right panel displays the loading plots (Figure 2B). Metabolites such as ornithine are positioned in the upper region of the loading plot. Correspondingly, the KO samples are in higher positions than the WT samples across all time points in the score plot, indicating consistently elevated ornithine levels in the KO mice. Metabolites such as isoleucine, glutamine, methionine, and phenylalanine are located in the lower region, exhibiting an opposite trend: their concentrations are consistently higher in the WT mice, regardless of time. Metabolites such as urea, located on the right side of the loading plot, align with the rightward shift of the samples over time in the score plot. These data suggest that the concentrations of these metabolites increase over time.

**Figure 3:**
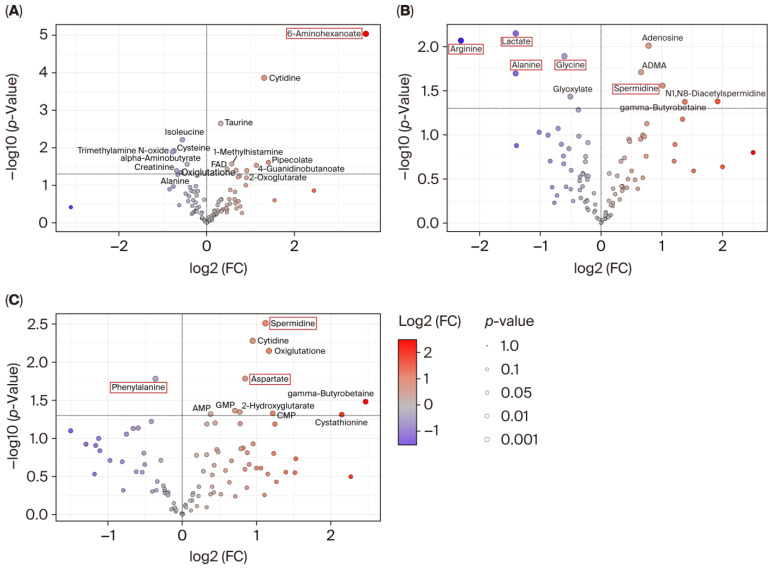
Volcano plots of metabolite concentrations. *X*-axis indicates log2 of (KO/WT). The *Y*-axis indicates −log10 (*p*-value). Metabolites showing significant differences (*p* < 0.05) are labeled. (**A**) 0 h, (**B**) 6 h, and (**C**) 18 h. We highlighted the compounds discussed in the text with red boxes.

The differences in the metabolomic profiles between the WT and KO groups are shown as volcano plots (Figure 3). At 0 h (Figure 3A), 6-aminohexanoate displayed a markedly high *Y*-axis value, corresponding to a highly significant *p*-value; however, its differential expression was not retained at 6 h (Figure 3B) or 18 h (Figure 3C), suggesting transient perturbation. At 6 h, several amino acids, including lactate, arginine, alanine, and glycine, appear on the left side of the volcano plot, indicating there are significantly lower concentrations in the KO mice compared to those in the WT.

In contrast, spermidine was upregulated in the KO mice, a trend that persisted through the 18 h mark. Moreover, at 18 h, the *Y*-axis value for spermidine further increased, reflecting an even smaller *p*-value and greater statistical significance.

Interestingly, phenylalanine, which showed no significant difference at earlier time points, was significantly downregulated in the KO mice at 18 h. Meanwhile, aspartate was significantly upregulated in the KO mice at 18 h, highlighting a dynamic shift in metabolite regulation over time. These findings underscore the evolving metabolic divergence between the WT and KO groups and suggest time-specific alterations in key metabolic pathways.

### 3.3. Metabolic Pathways

**Figure 4:**
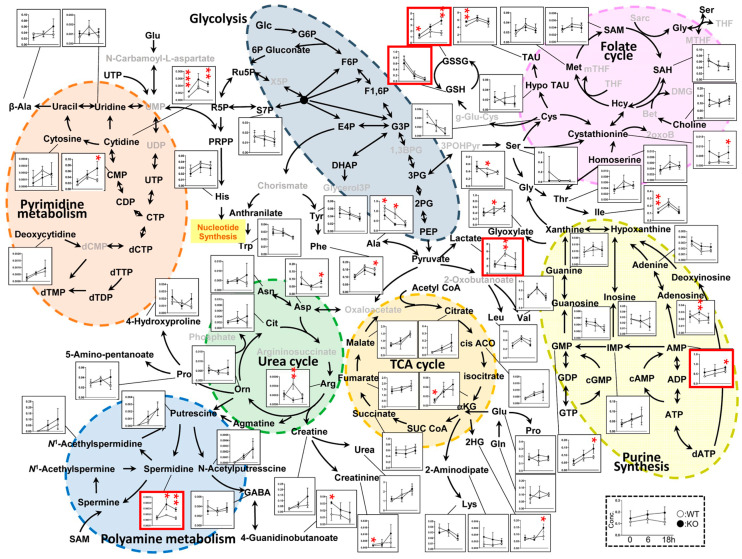
Metabolic pathway diagram. The filled (black) symbols represent the KO mice, and open symbols represent the WT mice. The *y*-axis indicates metabolite concentrations in μmol/g of liver tissue, and the *x*-axis shows time points: 0 h, 6 h, and 18 h after CLP. The metabolites enclosed in the red box in Figure 4 represent the representative metabolites shown in Figure 5: Lactate, AMP, GSH, GSSG, and Spermidine. Each data point and error bar represent the mean ± standard deviation (SD). Statistical comparisons between the WT and KO groups at each time point were performed using Student’s t-test. All data are expressed as means ± SD. Significance levels are indicated as follows: * *p* < 0.05, ** *p* < 0.01, and *** *p* < 0.001. Metabolites highlighted with red ellipses are those discussed in the main text.

Figure 4 provides a comprehensive overview of the metabolite concentrations measured in this study, encompassing key metabolic pathways such as glycolysis, the TCA cycle, the urea cycle, the pyrimidine and purine synthesis pathways, the folate cycle, and the polyamine pathway. Metabolites in the TCA cycle showed relatively minor differences between the WT and KO groups but generally increased in quantity over time in both genotypes. In contrast, the quantities of metabolites in the urea cycle tended to decrease over time, suggesting pathway-specific temporal regulation. Ornithine levels were significantly different between the WT and KO mice at 18 h; however, consistent with the heatmap and PCA results, the KO samples at this time point exhibited greater variability, as indicated by the longer error bars. In the purine synthesis pathway, there were differences in metabolites such as guanine, guanosine, and IMP between the WT and KO groups at 0 h; however, these differences diminished over time, indicating the convergence of purine metabolism as the condition progressed. The error bars for some metabolites appear unusually large. There are several reasons for this. One reason is the number of animals. In this experiment, we used nine mice for metabolomics in each group, so there were some variations, but this variation is biological. And the other is technical noise. If we had used more animals for metabolomics, the error bars might be smaller.

**Figure 5:**
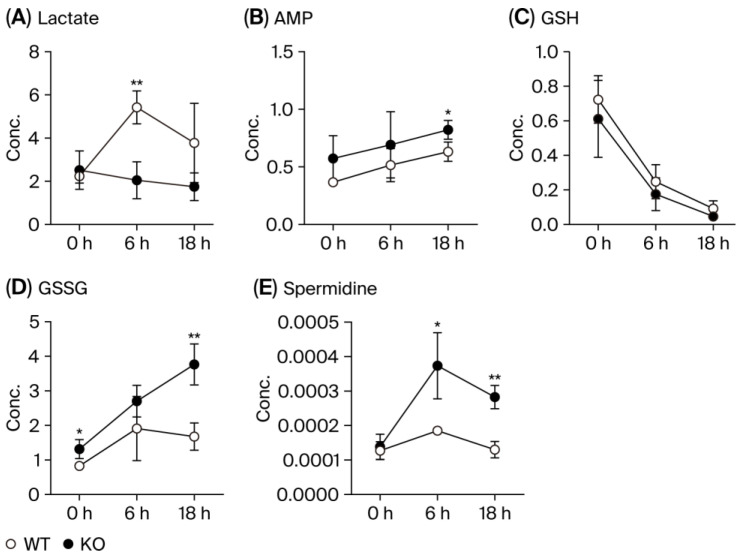
Time-course changes in representative metabolite concentrations. These panels show selected metabolites from the metabolic pathway presented in Figure 3: (**A**) lactate, (**B**) AMP, (**C**) reduced glutathione (GSH), (**D**) oxidized glutathione (GSSG), and (**E**) spermidine. All data are expressed as means ± SD. The significance levels are indicated as follows: * *p* < 0.05 and ** *p* < 0.01.

Figure 5 presents representative time-course data for the selected metabolites. Lactate, the end product of glycolysis, exhibited similar concentrations in both the WT and KO mice at 0 h. While the KO mice exhibited a gradual decrease in lactate levels over time, the WT mice showed a significant increase at 6 h. Although a difference remained at 18 h, the large error bars for the WT group prevented statistical significance from being reached. Levels of AMP, a breakdown product of ATP and ADP and a key indicator of cellular energy status, increased over time in both the WT and KO groups; however, the KO mice consistently exhibited higher average AMP levels, with a statistically significant difference observed at 18 h. Reduced glutathione (GSH), which plays a central role in maintaining cellular redox balance, showed a sharp depletion over time in both genotypes, with similar concentrations between the WT and KO mice. In contrast, oxidized glutathione (GSSG) levels increased over time, with the WT mice exhibiting a more pronounced rise and a significant difference from the KO mice at 18 h. Spermidine, an intermediate in the polyamine pathway, was present in comparable levels between the WT and KO at 0 h, but these levels were significantly elevated in the KO mice at 6 and 18 h.

### 3.4. MDA Production

**Figure 6:**
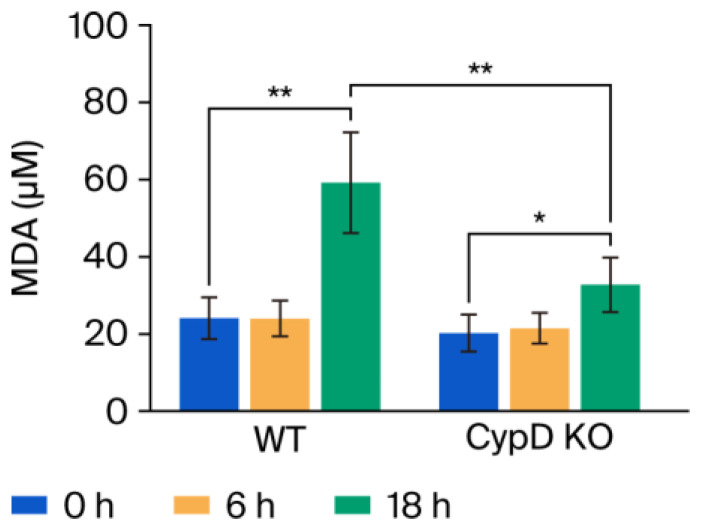
Comparison of MDA production between the WT and CypD KO mice. The *y*-axis indicates the concentration of MDA (μM) in mouse livers harvested 0, 6, and 18 h after CLP, quantified via the TBARS assay (*n* = 5). All data are expressed as means ± SD. The significance levels are indicated as follows: * *p* < 0.05 and ** *p* < 0.01.

MDA levels in mouse livers were quantified using the TBARS assay at 0, 6, and 18 h following CLP treatment and compared between the WT and CypD KO mice. At 0 and 6 h post-treatment, hepatic MDA levels were comparable between the WT and CypD KO groups. However, a significant increase was observed at 18 h in both genotypes, and the increase was significantly suppressed in CypD KO mice (1.62-fold) compared with WT mice (2.46-fold) (Figure 6).

### 3.5. Akt Phosphorylation

**Figure 7:**
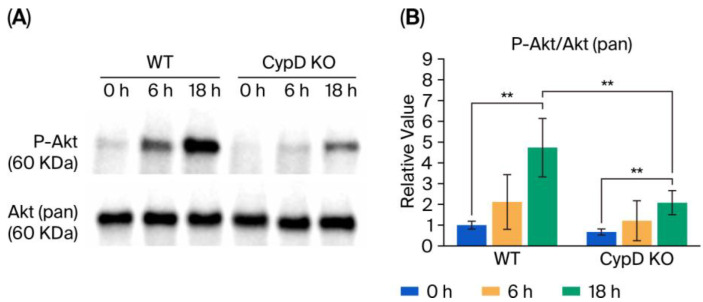
Comparison of Akt phosphorylation between the WT and CypD KO mice. (**A**) Western blot analysis of phosphorylated Akt (P-Akt) and Akt (pan) signals in WT and CypD KO mouse livers collected 0, 6, and 18 h after CLP. The original blot images are available in Supplementary Materials, Figure S1. (**B**) P-Akt/Akt (pan) ratio calculated from the quantitative WB signal (*n* = 6). The *y*-axis shows the relative value of the P-Akt/Akt (pan) ratio based on the WT value at time 0. All data are expressed as means ± SD. Significance levels are indicated as follows: ** *p* < 0.01.

Changes in phosphorylated Akt (P-Akt) and total Akt (pan-Akt) expression were examined using Western blot analysis in mouse livers collected 0, 6, and 18 h after CLP treatment (Figure 7A). The P-Akt/pan-Akt ratio was calculated based on a densitometric analysis of the blot signals. A significant increase in Akt phosphorylation was observed 18 h post-treatment in both the WT and CypD KO mice. Although individual variability was high and statistical significance was not reached, a trend toward increased phosphorylation was also noted at 6 h. Notably, CLP-induced Akt phosphorylation was attenuated in the CypD KO mice compared to that in their WT counterparts (Figure 7B).

## 4. Discussion

In this study, we focused on investigating the underlying metabolic mechanisms in the liver, a mitochondria-rich organ. Our primary analysis focused on metabolomics. Evaluating survival rates or body temperature was not directly demonstrated in the current work, nor was it our research objective. 

Metabolomic profiling revealed that the CypD KO mice maintained higher levels of AMP and exhibited significantly lower lactate accumulation compared to the WT mice. These findings suggest that mitochondrial ATP production was better preserved in the KO group, likely due to inhibition of MPTP opening. The suppression of lactate accumulation, a hallmark of anaerobic metabolism, further supports the notion that mitochondrial oxidative phosphorylation remained functional in the absence of CypD [7]. This metabolic preservation may help improve energy homeostasis and cellular viability under septic stress.

Oxidative stress is a major driver of organ dysfunction in sepsis [16], and our data indicate that CypD KO mice exhibit enhanced antioxidant capacity [4]. While GSH levels declined similarly in both groups, GSSG levels were significantly higher in the KO mice 18 h after CLP. This paradoxical increase in GSSG suggests that KO mice may have a more robust GSH turnover, enabling more effective scavenging of reactive oxygen species (ROS). On the other hand, the increase in GSSG relative to the decrease in GSH was more gradual in the wild-type group, suggesting possible inhibition of GSH-to-GSSG conversion or loss of glutathione. A decrease in the GSH/GSSG ratio is thought to indicate an imbalance in redox balance in the liver. The elevated GSSG levels, in conjunction with preserved AMP, suggest that mitochondrial redox buffering and ATP synthesis were maintained in the KO group, thereby contributing to cellular protection.

The original idea for this study came from our group’s previous work on ischemic neuronal cell death. Initially, cyclosporin A (CsA), an immunosuppressant and calcineurin inhibitor, was shown to be cerebroprotective with respect to ischemic and traumatic brain injury [9,17,18]. CsA binds to CypD, a component of the mitochondrial permeability transition pore (MPTP), and inhibits the opening of this pore [8,19,20,21]. CypD, which is encoded by the Ppif gene, is a peptidyl prolyl isomerase that is involved in forming the mitochondrial permeability transition pore [22]. CypD belongs to a family of enzymes called peptidyl prolyl isomerases that are present in the mitochondrial matrix and thought to be involved in the opening of the MPTP by serving as components of multiple protein complexes [22,23]. As previously reported, the CypDKO mice exhibited evidence of a brain-protective effect, as determined through comparison with the WT mice [4]. These results confirm there is a similar protective effect in the liver.

Polyamine metabolism also appeared to be modulated by CypD deletion. Levels of spermidine, a polyamine with known antioxidant and cytoprotective properties, were significantly elevated in the KO mice at 6 and 18 h, while putrescine levels remained unchanged [24,25,26]. The higher spermidine-to-putrescine ratio may reflect a shift toward enhanced resistance to oxidative stress and mitochondrial stabilization. Given that spermidine has been shown to activate mitochondrial trifunctional protein (MTP), a key enzyme in fatty acid β-oxidation [27], its elevation in KO mice may further support mitochondrial energy metabolism during sepsis. Another aspect of spermidine’s membrane-stabilizing action is its potential to regulate the opening of the mitochondrial membrane permeability transition pore (MPTP). The MPTP is a protein complex, and when it opens, it can lead to mitochondrial swelling and cell death. Several studies have suggested that spermidine may control MPTP opening and protect cells from mitochondrial-mediated cell death pathways [28,29].

The increase in the levels of MDA, an indicator of oxidative stress, observed 18 h after CLP administration was attenuated in the CypD KO mice compared to the WT mice, supporting the conclusion that CyPD KO suppressed oxidative stress. Western blot analysis revealed that the increase in the levels of P-Akt, a cell survival signal marker, was also suppressed in comparison to the WT mice. These results suggest that CypD deficiency may suppress the increase in oxidative stress levels caused by sepsis, and the increased spermidine levels revealed by metabolomic analysis may be one of the reasons.

Our results support the hypothesis that CypD deletion protects mitochondrial homeostasis by preventing the opening of the MPTP, thereby preserving ATP production and reducing oxidative stress. These data are consistent with the findings of prior studies, which showed that MPTP inhibition mitigates mitochondrial swelling, cytochrome c release, and apoptosis [8,30]. The observed metabolic shifts in the KO group—elevated AMP, GSSG, and spermidine levels, along with reduced lactate and MDA levels—collectively point to a more resilient mitochondrial phenotype under septic conditions. These metabolic signatures may serve as biomarkers for mitochondrial protection and therapeutic efficacy.

Despite these promising findings, our study has limitations. The metabolomic analysis was conducted on whole liver tissue, which includes hepatocytes as well as non-parenchymal cells such as Kupffer cells and endothelial cells. Thus, cell-type-specific contributions to the observed metabolic changes remain unclear. Future studies employing cell-specific analyses and pathway-targeted interventions are essential in order to fully elucidate the protective mechanisms of CypD deletion in sepsis.

In conclusion, our findings demonstrate that CypD deficiency confers metabolic and redox advantages in the liver during sepsis, likely through preservation of mitochondrial function and suppression of oxidative stress. These results highlight the therapeutic potential of targeting the MPTP and mitochondrial resilience pathways in the treatment of septic organ dysfunction.

## 5. Conclusions

In this study, we conducted a time-resolved metabolomic analysis of liver tissue from WT and CypD KO mice subjected to CLP, a clinically relevant model of sepsis. Our results revealed that CypD deficiency preserved mitochondrial function, as evidenced by the maintenance of AMP levels, suppression of lactate accumulation, and elevated concentrations of GSSG and spermidine. These metabolic signatures were accompanied by reduced oxidative stress, as indicated by lower MDA levels. Furthermore, principal component analysis and heatmap clustering demonstrated that the CypD KO mice exhibited distinct temporal metabolic trajectories and greater metabolic diversity at later time points, suggesting a more adaptive and resilient hepatic response to the septic insult. Collectively, these findings indicate that inhibition of mitochondrial permeability transition via CypD deletion confers protection against septic liver injury by maintaining energy metabolism, enhancing redox buffering capacity, and mitigating oxidative damage. Targeting mitochondrial permeability transition may thus represent a promising therapeutic strategy for improving organ function and survival in regard to sepsis.

## Data Availability

The original contributions presented in this study are included in the article. Further inquiries can be directed to the corresponding author, and it will be distributed upon request for transparency.

## Supplementary Materials

The following supporting information can be downloaded at https://www.mdpi.com/article/10.3390/metabo15090600/s1, Table S1: Figure 1 raw data, Figure S1: Original blots of Figure 7, Table S2: List of Abbreviations.

## Abbreviations

The following abbreviations are used in this manuscript: all abbreviations used in the figures are provided in Supplementary Materials, Table S2.

ADP	Adenosine Diphosphate
ATP	Adenosine Triphosphate
CLP	Cecal Ligation and Puncture
GSH	Reduced Glutathione
GSSG	Oxidized Glutathione
LC-MS	Liquid Chromatography–Mass Spectrometry
LC-TOFMS	Liquid Chromatography–Time-of-Flight Mass Spectrometry
MDA	Malondialdehyde
MPT	Mitochondrial Permeability Transition
MPTP	Mitochondrial Permeability Transition Pore
MTP	Mitochondrial Trifunctional Protein
PCA	Principal Component Analysis
ROS	Reactive Oxygen Species
TBARS	Thiobarbituric Acid Reactive Substances
TCA	Tricarboxylic Acid
TOF-MS	Time-of-Flight Mass Spectrometry
