# Supplementary figures and images for: Time-Resolved Metabolomics Reveals Mitochondrial Protection in Septic Liver Injury
Source: Metabolites. 2025 Sep 9;15(9):600. doi: 10.3390/metabo15090600 (PMC12472181; doi:10.3390/metabo15090600)

## Slide 1
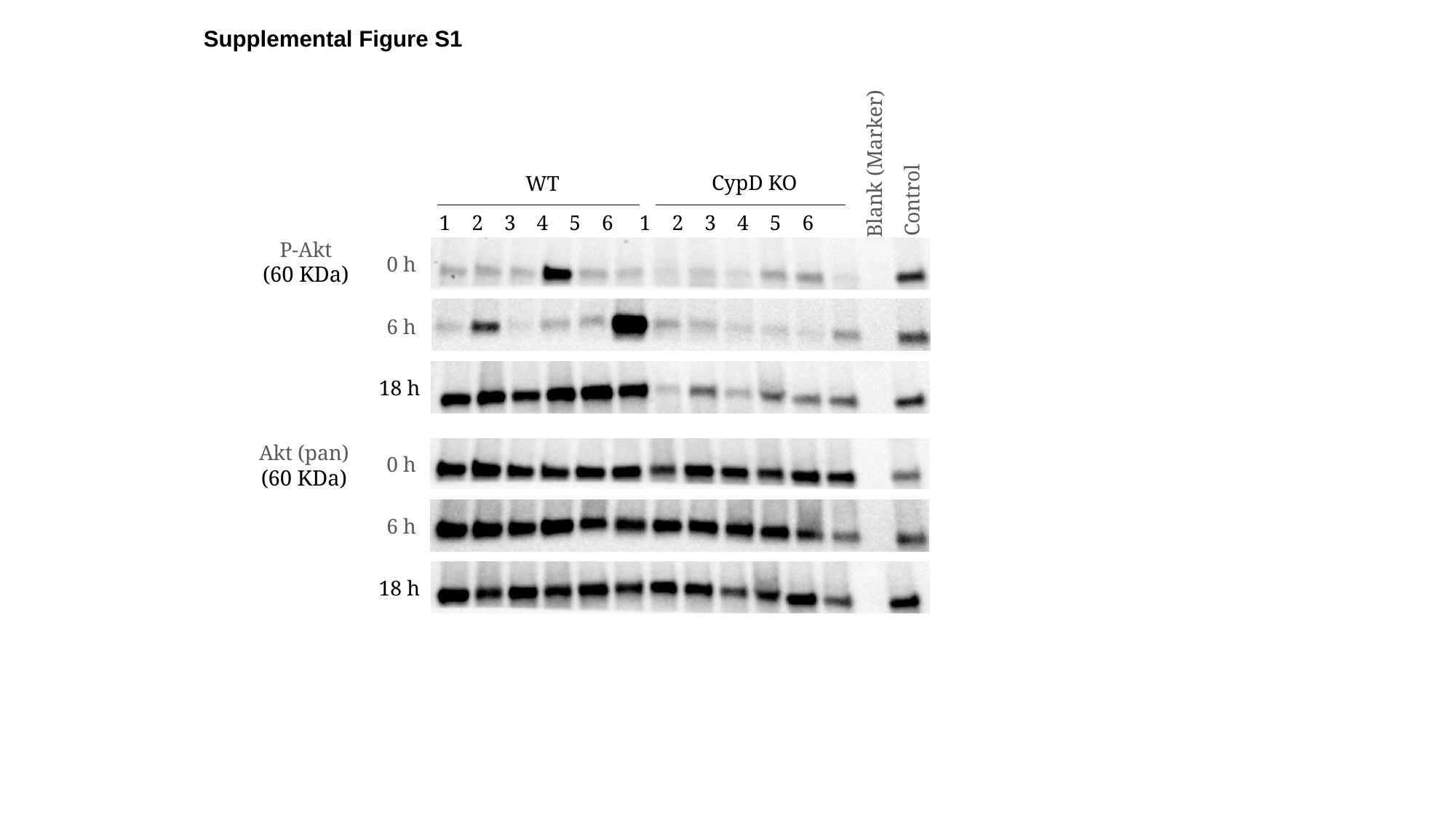

Supplemental Figure S1
Blank (Marker)
CypD KO
WT
Control
 1 2 3 4 5 6 1 2 3 4 5 6
P-Akt
(60 KDa)
0 h
6 h
18 h
Akt (pan)
(60 KDa)
0 h
6 h
18 h

Supplement: Supplementary file 1 [file metabolites-15-00600-s001.zip › Figure S1:Original blot of Figure7.pptx]
